# Current Impact and Long-Term Influence of the COVID-19 Pandemic on Iraqi Healthcare Systems: A Case Study

**DOI:** 10.3390/epidemiologia3040032

**Published:** 2022-09-29

**Authors:** Taysir Al Janabi, Sunny Chung

**Affiliations:** New York Institute of Technology College of Osteopathic Medicine (NYITCOM), Glen Head, NY 11545, USA

**Keywords:** COVID-19, public health, global health, health system, healthcare system, health policy, Iraq, pandemic response

## Abstract

Decades of wars, sanctions, and internal conflicts have compromised Iraq’s health system, which once was the best system in the region. National and international efforts to revitalize the system have been successful to some extent; however, significant challenges still exist. The COVID-19 pandemic has exposed new vulnerabilities and exacerbated the existing ones, affecting the quality and the quantity of the health services delivered. This case study explored the baseline function of Iraq’s health system within the context of the World Health Organization (WHO) health system framework. The paper also examined the country’s response to the COVID-19 pandemic and some of its impacts. Results show that the system was not functioning optimally nor was it prepared to address the immediate impact of the current pandemic and other emerging public health issues. While mitigating the pandemic’s short-term and long-term impacts are essential, it should not divert the focus from restructuring and strengthening the health system. Iraq may need to prioritize the health information system and leadership/governance as they provide the basis for health policies and regulations for all other health system building blocks.

## 1. Introduction

Significant achievements in health and healthcare systems have occurred in the past three decades, including upgrades in health infrastructure, improved health indicators, a decline in vaccine-preventable diseases, the use of information technology, and improved patient safety and inpatient quality of care. Despite these accomplishments, there have been many challenges, including a rise in non-communicable diseases, unequal distribution of healthcare facilities, and the uneven implementation and sustainability of health programs [1]. While health systems share the same purpose of protecting and improving health while ensuring the fairness and affordability of healthcare services, they are context specific. They can be interpreted differently regarding what constitutes part of the system [2]. Thus, addressing these challenges with just one set of solutions is not feasible.

The World Health Organization (WHO) established the health system framework in 2006, which has been fundamental in defining health systems, identifying areas of interest, establishing clear definitions of the global agency’s priorities, and recognizing gaps where assistance is needed. The framework consists of six blocks: service delivery; health workforce; health information; medical products, vaccines, and technologies; financing; and leadership and governance [3]. The framework has been essential in strengthening health systems and can be used as a medium for achieving national and global health targets [4].

Until 1980, Iraq had one of the strongest economies in the region, with revenue from oil production offering generous funding for development, resulting in impressive and solid health and education infrastructures [5,6]. Since then, Iraq has experienced years of war against Iran, forcing it to divert its resources from public services and civilian infrastructure, followed by the Iraq invasion of Kuwait. In response, the United States (US) led a global coalition against Iraq, which resulted in the Gulf War in 1991 and international sanctions, and eventually, the US-led invasion of Iraq in 2003 [6,7]. Subsequently, internal conflicts and political, economic, and security instability were the predominant contexts in Iraq. Thus, Iraq was the ideal candidate to benefit from the Millennium Development Goals (MDGs) and the Sustainable Development Goals (SDGs). In order to achieve some of the initiatives’ goals, Iraq needed to reform the clinical and administrative aspects of its health system. The use of the WHO health system framework would be beneficial in assessing and strengthening Iraq’s health system [8].

Since the 1918–1919 influenza pandemic during the beginning of the last century, the COVID-19 pandemic is only the second outbreak to significantly influence all countries’ healthcare systems and economies, and the psychology of the world’s population [9,10,11]. The current pandemic has posed unique challenges to all health systems, regardless of their income level, as countries struggled to minimize their mortality and morbidity [12]. However, high-income countries have generally been more equipped with readily available resources to fight this pandemic [13]. Countries with low–middle income with suboptimal functioning health systems have been likely to endure more negative impacts of the pandemic [12].

This paper’s purpose is to describe Iraq’s health system in the pre-COVID-19 era within the context of the WHO health system framework, highlight the government’s response to the current pandemic, and identify some of the lessons learned that could improve essential health services in the short term and lessen the consequences of health emergencies in the future.

## 2. Materials and Methods

### 2.1. Search Strategy

This paper is a qualitative analysis involving a comprehensive review of the academic literature, official government documents, and reports from credible national and international organizations. The search was performed in June 2022 and included studies published from the beginning date of each search to August 2022. The literature search was conducted in PubMed, Cochrane Library, and the WHO (see Table A1). The Cochrane database was searched for grey literature. A search was performed using the focused key medical subject heading (MeSH) terms for: “SARS-CoV-2”, “Iraq”, and “global public health.” The same search strategy from PubMed was applied to Cochrane Library (see Table A2 and Table A3). Results were imported into Endnote and deduplicated by an independent reviewer. A comprehensive review of the official website of the Government of Iraq was conducted to identify policies related to the COVID-19 response. From the PubMed search, 379 results were found. Additionally, eleven and 59 results were found from the Cochrane Library and WHO search, respectively. After deduplication, 390 results remained. Researchers then filtered in Endnote for “COVID-19” and “global health”, and this search resulted in 154 articles. Details of the search method are shown in Appendix A. Due to the nature of this study, approval by the Institutional Review Board was not required.

### 2.2. Data Extraction

An extensive desk review of the literature on health systems, COVID-19 epidemiology and impact, government response, nonpharmaceutical intervention, vaccine acceptance, and the Iraqi context was conducted. In terms of sources, the authors used reports and policy briefs from the United Nations (UN) and several of its agencies and missions in Iraq: WHO, United Nations Children’s Fund (UNICEF), United Nations High Commissioner for Refugees (UNHCR), and United Nations Assistance Mission for Iraq (UNAMI). These documents provided a description of the health system and its strengths and weaknesses and provided an overview of the impact of COVID-19. The official website of the Government of Iraq and its ministries (Ministry of Health and Ministry of Planning) were reviewed, and official documents were also examined for the latest assessments and plans. The authors also reviewed the relevant academic journals and international and national organizations’ reports (The World Bank, United States Agency for International Development, KAPITA) as they provide a more in-depth assessment of government and health sector responses to COVID-19 and are more likely to report on some of the emerging public health issues. Qualitative data were mostly extracted to align with the narrative nature of the study; however, quantitative data were incorporated to supplement the narrative synthesis and discussion.

## 3. Case study

### 3.1. Case Presentation

To provide a comprehensive overview of the base functioning level of Iraq’s health system, the paper describes the Iraqi health system within the WHO’s health system framework.

#### 3.1.1. Service Delivery

**Figure 1:**
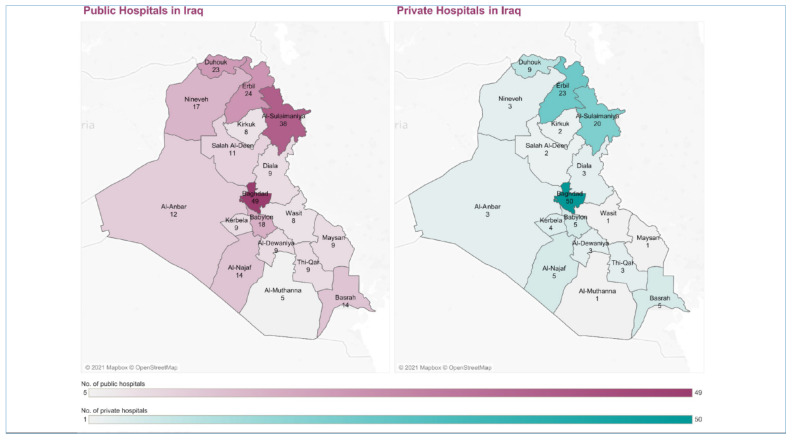
The number and distribution of the public and private hospitals in Iraq in 2019 (permission to publish this figure was granted from KAPITA).

**Figure 2:**
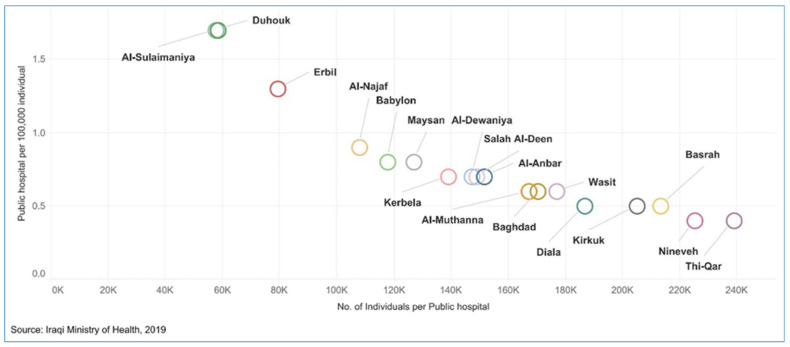
The number of the public hospitals per 100,000 individuals in Iraq in 2019 (permission to publish this figure was granted from KAPITA).

Health services are mainly provided by the public sector and managed centrally by the Federal Ministry of Health and Environment (hereafter called the Ministry of Health), which manages 19 provincial health departments (one in each province and two in the Capital, Baghdad). In 2022, the public sector provides services at a low cost through a network of 281 public hospitals and 2765 primary health centers (PHCs) [14]; each center serves 10,000–45,000 people [15]. The number of health facilities has improved since 2012. However, the number and the distribution of these facilities are inadequate as they are concentrated in Baghdad (see Figure 1 and Figure 2) [6,14]. Additionally, this increase has not met the heightened demand because of population growth. Moreover, about half of the PHCs have only one medical doctor; nurses and other health professionals manage the rest. While it is not uncommon for specialized health professionals and hospitals to be in major cities, population density and health needs should be considered in order to achieve equitable distribution of health facilities and human resources [16]. Iraq has 1.3 beds per 1000 individuals and fewer than 1000 intensive care unit beds in total [17,18].

Primary health care was introduced to the health system four decades ago, but the dominant health care was hospital-focused, resulting in the negligence of PHCs. The health services in Iraq have often been described as poor quality with long waiting times [16]. Additionally, limited medications, a shortage of skilled health workers, and pervasive corruption within the health sector reduced accessibility to health services [16,19]. Furthermore, the hospital-based model has been expensive to maintain. The Ministry of Health has now shifted health care to be more focused on primary care with the utilization of PHCs [6]. Mental health services are included in the primary health care package [6]. The next level of care is a referral from the PHCs to the referral hospital; however, only 40% of people get access to their referral services [6,20].

Unpaved routes in rural areas and limited ambulances might limit the transport and connectivity with health facilities; it takes on average more than 20 min for a family to reach the nearest PHC, and it takes about 32 min in rural areas [21]. According to the World Bank’s Systematic Country Diagnostic (SCD) of Iraq in 2017, fewer than 20% of Iraqis were satisfied with their health services [22,23]. Dual practice is a common phenomenon in Iraq where physicians work in the private sector while being full-time employees of the Ministry of Health. Conflicts of interest could be an issue of dual practice. The dual practice has neither been evaluated nor regulated.

Despite the unequal contribution and distribution of the private sector (see Figure 1), it still provides health services to those who can pay out of their own pocket, which is beyond the purchasing power of many Iraqis. Additionally, this sector has not evolved as an independent medical practice because it is under the influence of a minority of political figures who dominate the public health sector [19]. Moreover, the private sector lacks a national regulatory framework.

The Kurdistan Regional Government (KRG), a semi-autonomous regional government known in the north of Iraq, has its own regional Ministry of Health, established in 1992 with a structure and character similar to the Federal Ministry of Health; both are described as politicized with a lack of transparency and regulatory and accountability frameworks [24]. However, the KRG has a better and more stable health system than the rest of Iraq because of its political stability, relatively stable security, and the expansion of its private sector [22].

#### 3.1.2. Health Workforce

Developing and retaining the health workforce is one of the challenges the health system faces in Iraq. The country is rich in human resources with a network of medical, nursing, and paramedical colleges; the Ministry of Higher Education and Scientific Research is the main provider of medical doctors with 27 medical colleges [21,25], while the Ministry of Health is responsible for training nurses and midwives in its nursing and midwifery high schools. The number of medical doctors and nurses per 10,000 population in 2020 was reported to be 9.66 and 23.87, respectively, which was lower than Iraq’s neighboring countries except for Iran, where the number of nurses was comparable [26]. These human resources are concentrated in Baghdad, leaving other governorates with even lower numbers of doctors and allied health professionals. Many older doctors and consultants received training in the United Kingdom (UK). However, they remained out of the international medical community because of wars and sanctions, leaving them relying on outdated equipment and practices [27]. Many health professionals were killed or forced to leave the country because of life-threatening situations.

In 2013, it was reported that about 25% of new medical graduates left the country for better personal and professional opportunities. Attempts to bring back Iraqi doctors have been unsuccessful. Doctors, nurses, and other health professionals are Ministry of Health employees. However, their distribution to different health facilities depends on many factors, including personal preference, proximity to family, and opportunities for private practice. These factors contribute to a disproportionate doctor/nurse ratio and unequal distribution of human resources [6,21]. Incentives, whether monetary or professional, may encourage the recruitment and retention of health professionals in remote and rural areas. After 2003, a nursing training strategy was adopted to train nurses to earn a diploma or college degree; the latest report in 2017 stated that only 30% of nurses were educated to that level [6,28].

#### 3.1.3. Health Information

The collection, analysis, and dissemination of health information is crucial in policy development, health system management, and monitoring and evaluating epidemiological and demographical concerns [8,16]. In 2019, Iraq’s health information system had only 24% of the elements that constitute a fully functional and operational information system [15]. Additionally, the service delivery system and the health information subsystems are fragmented [29]. Health information, mainly in paper form, typically flows from the periphery to the center, from health facilities and district health offices to the governorate Directorate of Health, and finally to the Ministry of Health. Electronic upgrades in data collection and transfer are still in the early stages. Establishing a unified health information system that integrates information subsystems has been a priority of the National Development Plan 2014–2018 and National Health Policy 2014–2023 [21,30,31]. A surveillance system for vaccine-preventable diseases is part of the health information system that successfully reports data on time, but overreporting concerns exist [16].

#### 3.1.4. Medical Products, Vaccines, and Technologies

The Directorate of Technical Affairs within the Ministry of Health is the only medicinal regulatory authority in Iraq. Approving medicines and vaccines, issuing marketing authorization, and quality control and assurance are a few of its responsibilities. The National Committee for Drugs Selection (NCDS), which is part of the Directorate of Technical Affairs, is responsible for selecting medicines and vaccines for the nation [32].

The country has traditionally been dependent on large-scale imports of medicines and equipment [33]. Until the US invasion in 2003, the state-run marketing drugs and medical devices company Kimadia was the only importer and distributor of medicines, equipment, and health-related technologies to the public sector. The government expenditure on pharmaceuticals was USD 1.25 billion in 2019, representing 25% of the total health expenditure [32,34]. Kimadia is required to procure specific drugs and vaccines recommended by the NCDS, while the rest of the medications can be procured by the private sector [32]. A lack of adequate supply and delays in delivery are the predominant features of Kimadia because of financial, operational, and logistical reasons.

Since 2003, there have been robust private pharmaceutical companies with about 1000 outlets; however, these companies’ work remains largely unregulated, resulting in substandard and falsified medications in the market [6,21]. In 2014, the counterfeit pharmaceutical industry was estimated to generate USD 1 billion a year, which was one-fifth of all pharmaceutical sales in the country [35].

#### 3.1.5. Health Financing

The first national health account was established in 2010 when 8.4% of Iraq’s gross domestic product (GDP) of USD 82.2 billion was spent on health, later reported to be 4.5% of its GDP of USD 110.83 billion in 2019. The percentage is low compared to the other Middle Eastern countries with comparable GDPs [6,32]. The Ministry of Health is the significant health sector funder. In 2012, the government contributed 59% through the public sector, while the private sector contributed 41%. More than 47% of the government health expenditure was for the salaries of the health workforce, and 30% was for pharmaceutical products [16]. In 2019, the health expenditure per capita was USD 253.3, which had improved from USD 150.5 in 2010, but it was still far lower than Iraq’s neighboring countries [36].

#### 3.1.6. Leadership and Governance

The leadership and governance block is another critical building block as it provides the basis for regulations for all other health system blocks [8]. The lack of effective leadership is a major contributing factor to health system failures in low- and middle-income countries (LMICs) [37]. Widespread corruption has led to the loss of financial resources for the health sector, undermined the leadership of many of the health institutions, and undermined public confidence and trust in the health system; many individuals in leadership positions are unqualified or under pressure from their affiliated political entities to employ specific agendas [34]. A revised National Health Policy was supported by the WHO in 2015—a policy that was set up to reshape the health system for the next decade—with an emphasis on improving the building blocks of the WHO health system framework [38]. A wide range of domestic and overseas trainings have been put in place to improve leadership and build management capacity at the Federal Ministry of Health and its Directorate of Health.

### 3.2. COVID-19 Case Importation

On 28 January 2020, Iraq announced specific measures to prevent the entry of SARS-CoV-2 into the country: evacuating citizens from Wuhan, China, checking temperatures, and quarantining symptomatic patients at the border; Iraq acted proactively in response to the novel coronavirus outbreak, even when its neighboring countries reported no cases. Additionally, the government established the Government Crisis Cell to coordinate its health-related response to COVID-19 under the guidance of the Minister of Health [39].

The first COVID-19 case confirmed in Iraq was in Najaf, about 112 miles south of Baghdad, on 24 February 2020—an Iranian student who had gained entry to the country before the entry ban on Iranian nationals became effective [40]. In contrast, the first COVID-19 case in Kurdistan was detected on 1 March 2020. The case was also a returnee from Iran. Many Kurdistan residents travel to Iran for various reasons such as medical treatment, tourism, business, and studying at Iran’s universities [41]. However, concerns remained around people crossing through unofficial routes and bypassing inspection at the border [42].

### 3.3. Management and Outcomes of COVID-19 in Iraq

The government established the Iraqi Higher Committee for Health and National Safety in March 2020 to direct the national efforts in the fight against COVID-19. The committee included several ministers, the Governor of the Central Bank of Iraq, the National Security Adviser, and other officials to address the impact of the pandemic on different aspects of life [43]. By the time the WHO declared the COVID-19 pandemic on 11 March 2020, Iraq had only 61 confirmed cases and six deaths [44].

On 17 March, the committee announced total lockdown measures, which remained in place until 21 April 2020; these included closing all public and private organizations, and academic and educational institutes, and restricting transportation and mobility between cities [45]. Despite the lockdown measures, pilgrims defied these restrictions to commemorate the anniversary of the death of revered Shiite Imam Musa Al-Kadhim on 18 March 2020. Health authorities had reported 13 deaths and 164 infections from the virus by that time, reaching up to 50 deaths and 694 two weeks after the event. However, many suspect the number of cases could have been higher and unnoticed because of the limited testing capacity at the time [46]. The KRG implemented similar measures; however, it was stricter as it ordered people to stay home and banned any mass gatherings, including Newroz—an annual festival that marks the beginning of a new year among many ethnic groups including Kurdish people, celebrated from 21 March to 1 April [42,47,48].

Initially, all specimens were processed by the Ministry of Health laboratory in Baghdad, which made it challenging to process specimens from rural areas. Additionally, the lack of skills and equipment to obtain these specimens forced suspected patients to be referred to major hospitals in Baghdad. With no referral protocol in place, the risk of transmission was significant [49]. With the support of China and the WHO, the Ministry of Health opened three molecular laboratories (one each in the northern, central, and southern regions of Iraq) for COVID-19 testing, which increased the country’s testing capacity to 100 tests per day [46]. Iraqi returnees from specific countries were offered polymerase chain reaction (PCR) tests and asked to self-quarantine until testing results were available. However, many Iraqis live in multigenerational homes with people of varying ages and vulnerabilities, and many others could not afford to self-isolate because of housing and financial barriers. Additionally, PCR results initially took more than two weeks to come back, hindering their purpose.

From April 2020 until September 2020, a partial lockdown was imposed in which travel was prohibited between 8 PM and 5 AM, shops and businesses were closed, and vehicles were only allowed on roads on alternating days based on license plate number (even or odd) [50,51]. Two further periods of complete lockdown were imposed: one during Eid al-Fitr (a religious holiday that marks the end of Ramadan, the Muslim holy month of fasting), and the other during Eid al-Adha (another religious holiday that marks the end of Hajj, the pilgrimage to Mecca for Muslims) for 15 and 10 days, respectively [45]. Most religious holiday activities involve indoor social gatherings with relatives and friends, which may occur in the same neighborhood and are difficult to restrict.

**Figure 3:**
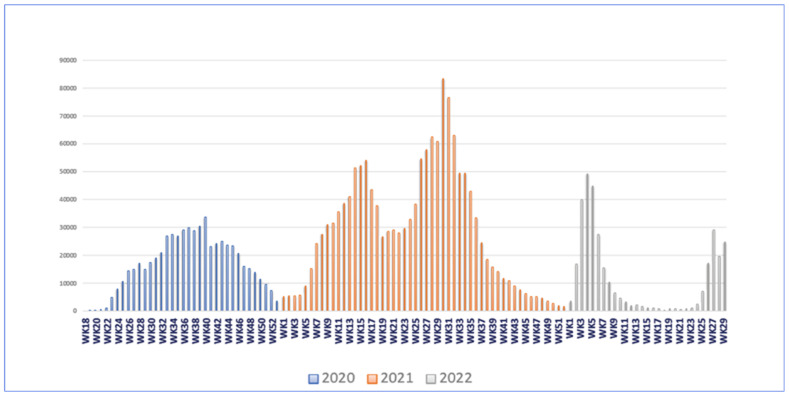
COVID-19 epi curve by weeks in Iraq 2020–2022. The graph was adapted from Iraq’s situation report as of 24 July 2022, by WHO.

From September 2020 to February 2021, the lockdown was lifted entirely in Iraq. However, a partial lockdown was then reimposed in February 2021 when the second wave of the pandemic started due to inconsistency in implementing protective measures and more people being confined in their homes during cold weather [42]. Despite these restrictions, the daily cases gradually increased and peaked on 28 July 2021 when there were 13,515 confirmed cases, and the total number of deaths was 9301 (see Figure 3) [44].

**Figure 4:**
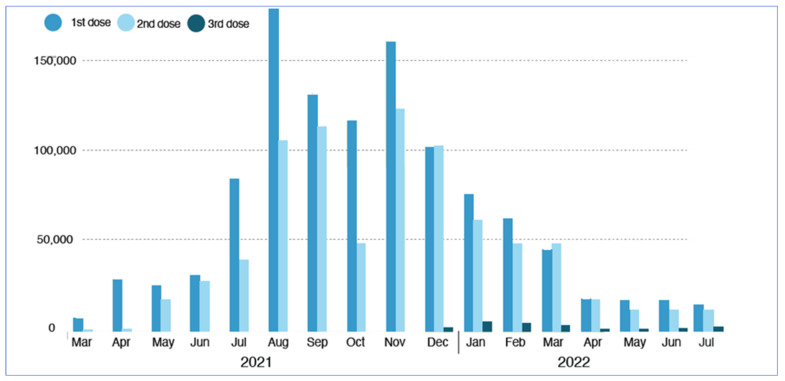
Monthly COVID-19 vaccination by doses in Iraq 2021–2022. The graph was adapted from Iraq’s situation report as of 24 July 2022, by WHO.

Iraq received its first COVID-19 vaccine shipment of 336,000 doses of AstraZeneca at the end of March 2021, with an additional 1.1 million doses arriving in the following weeks [52]. According to the national vaccine deployment plan, the Ministry of Health distributed vaccines to health institutions nationwide to be used first for priority groups (healthcare workers, military personnel, and the elderly). By July 2021, only 7.4% of the targeted Iraqis were vaccinated, while the second highest cumulative number of confirmed cases of 1,421,746 and the third highest number of total deaths in the region was reported [53,54]. While Iraq kept reporting the second highest number of cases in the region, the country commenced its massive vaccination campaign in November 2021 to align with the WHO’s global vision of achieving 40% vaccine coverage by the end of the year [55,56]. Iraq established more than 100 vaccination sites across the country to enhance vaccination after it was reported that only 9% were fully vaccinated [57,58]. About 11 million Iraqis had received at least one shot of the COVID-19 vaccine by the end of July 2022, representing more than 25% of the population (see Figure 4) [58,59].

The acceptance of the COVID-19 vaccine was low in the countries of the Middle East. For example, COVID-19 vaccine acceptance in Iran was reported to be less than 20% in 2021 [60]. A study conducted in December 2020 to explore willingness to accept the COVID-19 vaccine among Arab countries (Jordan, Lebanon, Kingdom of Saudi Arabia, and Iraq) reported that vaccine acceptance was 17.1%, 18.5%, 29.4%, and 34.7%, respectively [61]. Most of the population in the Middle East knew that COVID-19 was a reality, but two-thirds of the population believed that the disease was a biological weapon; such belief has impacted public adherence to preventive measures [62,63,64]. This variation in vaccine acceptance could be attributed to the local COVID-19 prevalence and mortality rates, the countries’ economic, social, and political status, education, national awareness, and most importantly, the public’s trust in their government and health authorities [61].

Confirmed COVID-19 cases in Iraq were required by law to be admitted to the Ministry of Health quarantine facilities for at least ten days and would be discharged after two negative PCR tests [65], as there were 22 quarantine centers across the country. Admitted patients at these quarantine facilities received hydroxychloroquine and azithromycin; this treatment protocol was approved by the Iraqi Scientific Committee on 1 March 2020 [66]. Contacts were required to quarantine in designated health facilities for observation; however, with increasing cases beyond the quarantine centers’ capacity, contacts were asked to stay at home. Contact tracing was implemented by rapid response teams distributed in all districts, which included daily home visits with temperature and respiratory symptoms checks [67]. Additionally, active surveillance began in early May 2020; teams would look for suspicious cases in 50 houses around the index case of an area, then followed up with rapid testing for suspected cases. If a rapid test came back positive, then a PCR test would follow. However, rapid testing kits from different local and international sources were used before assessing their validity and the specimens’ processing time was long [67].

Later, mild to moderate severity patients were asked to isolate at home [67]. The exponential increase in cases occurred in June 2020 when cases were reported to be more than 1000 per day [66]. Subsequently, there was a general shift in management where cases of different severity were to be managed by private practitioners at home [67]. Individuals procured their own oxygen tanks and set up home-based care, which could be unsafe and costly amid an oxygen shortage in the country [19]. To urgently address the oxygen crisis, oxygen supply was purchased from Kuwait [67]. Additionally, the WHO provided Iraq with 300 oxygen concentrators [68].

Economically, Iraq implemented specific measures to mitigate the economic impact of the pandemic. First, the government halted deductions from its employees’ salaries to repay loans, residential rentals, and mortgages. These measures primarily targeted public sector employees, leaving the daily wage workers and other vulnerable segments of society unsupported financially [69]. Later, the government allocated temporary financial assistance for the most affected individuals; to qualify, an individual had to sign up online via a government-based website. However, this measure did not benefit those affected the most as most of those individuals lacked internet access, were computer illiterate, and the signup was only available for five days. Finally, the prices of food and medical supplies were also monitored [67]. It was estimated that 7 million out of 12 million eligible individuals registered for temporary financial assistance [70].

The government also increased internet capacity to accommodate the new reality of the virtual world as many governmental and educational entities and several businesses transformed their activities [69,71]; however, only 48.3% of the population have internet access [72]. Education witnessed a major transformational phase in response to the pandemic, but the lack of expertise, poor internet service, and content quality were the main challenges [73,74,75]. Concerns regarding online learning widening the education gap between poor versus rich students and urban versus rural also emerged [75,76].

### 3.4. Current Impact and Long-Term Influence of the COVID-19 Pandemic on Global and Public Health and Healthcare System

#### 3.4.1. Vaccine-Preventable Diseases (VPDs)

Iraq’s immunization strategy is based on three pillars: administer vaccines, conduct national immunization campaigns, and maintain surveillance. Iraq successfully eliminated polio in 2000, validated its maternal and neonatal tetanus elimination with WHO and UNICEF, increased vaccine storage capacity, and developed a comprehensive Expanded Program on Immunization (EPI) [38,77]. Iraq also introduced Haemophilus influenzae type b (H. influenzae) and rotavirus vaccines in 2012 and the injectable polio vaccine in 2015. Additionally, Iraq replaced the trivalent oral polio vaccine with a bivalent oral polio vaccine in 2016 [38].

A study that assessed vaccine uptake in children under the age of 5 years prior to, during an early phase, and in the later phase of the current pandemic for the following vaccines: Bacille Calmette-Guerin (BCG), pentavalent (diphtheria, tetanus, pertussis + H. influenzae type b + inactivated polio vaccine) doses 1, 2, 3, measles, mumps, and rubella (MMR), and activated oral polio vaccine doses 1, 2. The findings showed a dramatic decrease in vaccination during the pandemic, especially for MMR; the rate of MMR uptake was 83.7% in 2018, 69.1% in 2019, and 63.6% in 2020 [78]. Additionally, available data show that vaccine-preventable diseases have occurred at an increased incidence and caused outbreaks because of the fluctuation in vaccination coverage, which was reported to be between 60–80% [79]. Fourteen governorates reported more than a 10% drop-out between diphtheria, tetanus, pertussis vaccine (DTP) dose 1 and 3 in 2014, which was higher than the maximum acceptable rate [80].

In April 2020, UNICEF and the WHO called on the Iraq government to increase its investment in health services and target children who had missed their immunization because of the pandemic [78]. Iraq remains at high risk for importing wild poliovirus and vaccine-derived polioviruses because of the high internal and external population mobility, relatively low routine immunization coverage, and limited access to health care in some areas [81].

#### 3.4.2. Violence

##### Gender-Based Violence (GBV)

GBV is a global and human rights issue that has worsened during the pandemic, which has exposed the true extent of gender inequalities worldwide and created suitable circumstances for GBV to rise [82]. The pandemic has also exacerbated the pre-existing GBV in Iraq. The Family Health Survey conducted in 2006 showed that Iraqi women experienced different types of GBV; the most common forms were emotional and physical abuse [83]; in 2013, a study reported that the prevalence of GBV in Iraq was 45% [84]. Reports of GBV increased during the lockdowns, with a prevalence of 32% in Kurdistan; however, the number of reports might be underestimated because of the closure of the crisis offices, the presence of the abuser and the victim at home all the time, and the prioritization of other resources to respond to the pandemic [83]. Initial rapid assessment exercises conducted by the Health Cluster in Iraq indicated a sharp increase in health service utilization by GBV survivors. Forty percent of health service providers indicated an increase in survivors seeking help from rape, the sexual harassment of minors, and suicide related to intimate partner violence [85].

##### Violence against Health Care Providers (HCPs)

Violence against Iraqi HCPs is not a pandemic-generated issue. Death from intentional violence against Iraqi HCPs increased gradually from 10.6/1000/year in 2003 to a peak of 47.6 per 1000/year in 2006 [86]. A study about immigrated Iraqi doctors in Jordan in 2010 showed that 61% of 401 doctors were victims of violence, while 75% of the same participants reported that their families were targeted [87]. In 2018, another study showed that 85% of Iraqi HCPs in Baghdad were exposed to violence in the workplace. Most participants cited that the poor quality of health services and limited medications were contributing factors. Additionally, there was mutual mistrust between the HCPs and patients [88].

Violence against HCPs has increased since the pandemic as 88.3% of the 505 surveyed doctors reported physical and verbal violence in the prior six months. Overcrowded hospitals and the risk of contracting COVID-19 infections from health facilities were cited as reasons for violence [67,88]. It was also reported that these incidents were underreported because of the low expectations of HCPs that these events would be addressed by health officials [88].

#### 3.4.3. Mental Health

Healthcare workers (HCWs) reported higher levels of stress because of the high workload, putting themselves at risk, and concerns of spreading infection to their families and friends [89]. HCWs from Iraq were more likely to report anxiety, depression, and stress than others in the Middle East region. Additionally, doctors reported that they were stigmatized (people were avoiding them because of their work) [90]. Moreover, a survey exploring violence against medical doctors in Iraq showed that more than 50% of them did not receive adequate support from their health facilities, such as personal protective equipment (PPE). About 50% of the participant physicians felt appreciated by health officials, and fewer reported that they were appreciated by society [91]. A study that explored the perceived stress during the early stages of the pandemic in Iraqi Kurdistan showed that most medical specialties had perceived moderate levels of stress; the highest stress was among general physicians and medical lab specialists. The perceived stress was significantly higher among female doctors than their male counterparts [92,93]. Additionally, all doctors reported a high level of stress, irrespective of whether their field was related to COVID-19 or not [92].

The pandemic’s impact on the mental health of older populations was also evident in different contexts because of their isolation, being away from their caregivers, and exposure to new measures to adjust to life with limited support. Seventy four percent of older people felt worried or anxious, 68% felt depressed about the situation, and 48% depended on external support to cope [85]. Additionally, the pandemic hindered access to medications for chronic health issues and thus exacerbated the mental challenges in the older population. About 32% of the Iraqi older population did not have access to medications at the start of the pandemic [85].

#### 3.4.4. Impact of COVID-19 on Elective Medical Procedures

The pandemic posed a significant challenge to the organization of the health system as more resources were diverted from non-emergency clinical cases to more urgent ones. For example, elective surgeries were postponed and new protocols were implemented to minimize exposure [94]. The Neurosurgery Teaching Hospital (NTH) in Baghdad, which serves about 50% of the population in the capital, took specific measures to contain the spread of COVID-19 among its staff and patients. Admission to the hospital was limited to emergency cases, which were admitted directly, and to urgent cases, whose admission was determined by the hospital committee consisting of three neurosurgeons. Upon admission, patients underwent temperature and exposure screening. A PCR test was offered to those who were symptomatic [95]. Full PPEs were provided to the staff that worked in the neurosurgical intensive care unit (NICU) and those in contact with COVID-19 patients; the rest of the medical staff were provided with only surgical masks. Most of the patients were brought to the hospital by their relatives, who often defied the preventive measures at hospital entry. Additionally, the limited disinfection supplies challenged any efforts to prevent cross-contamination in the hospital. It was reported that 16% of the neurosurgical staff had been infected with COVID-19 by November 2020 [95].

When surgery was deemed to be necessary, the following intraoperative procedures were taken to minimize exposure: craniotomy without bone drilling, limiting the use of microscopes, endoscopes, and surgical chairs, restricting the number of personnel in the operating room, and only senior neurosurgeons were allowed to perform surgeries. The operating room was disinfected, ventilated, and isolated between surgeries for 24–48 h. Patients were discharged soon after the procedure and followed through with regular phone calls; patients were discharged two days after craniotomy and one day after a spine procedure, both of which were reduced from eight and four days, respectively, from before the pandemic. The total number of operations from January–July 2020 was reduced by 61.7% from the same period in 2019 because of the curfew and reduced referrals from other governorates [95]. Significant reductions in the utilization of healthcare services were reported in the first half of 2020 worldwide. Doctor’s visits fell by 42% while admissions, diagnostics, and therapeutics dropped by 28%, 31%, and 30%, respectively [96].

Kidney transplant procedures are performed at eight renal transplant units in Iraq, with the largest center being the Nephrology and Renal Transplantation Center, a governmental facility in Baghdad. The transplant activities, in general, ceased from February to May 2020, with a few procedures conducted in Kurdistan [33]. For dialysis patients, the Al-Zohoor hemodialysis center in the Central Teaching Hospital took specific measures to minimize exposure to COVID-19. For example, health education was provided to both the medical staff and the patients. Patients were encouraged to discuss their symptoms over the phone if they did not feel well before coming to the unit. Patients were also advised to use private transportation to limit exposure and to be accompanied by only one caregiver, who should stay in the waiting room wearing a disposable head cover, face mask, and shoe covers. Limiting eating, drinking, and talking in both the waiting and the dialysis room were also advised. Limiting the movement of the staff in and out of the dialysis units was implemented. Each patient used the same dialysis machine when they arrived at the center; extensive disinfection was implemented between shifts and after each patient’s sessions. With the abovementioned measures, it was reported that only three dialysis patients had tested positive for COVID-19 by 15 August 2020 [97].

## 4. Discussion

Iraq and its citizens have been in crisis for the most part since the country’s independence in 1921. The political situation and economy have always been in turmoil, which is reflected in the state of the health system, which can be described as an emergency-based system [87,98] that often focuses on short-term outcomes with the goal of minimizing damage instead of improving and promoting health. The COVID-19 pandemic has exacerbated the political, economic, and health divide within and among countries. Iraq with its shattered background is no exception.

The country has all the components that constitute a health system, but each element is defective and suboptimal, and since these blocks are interconnected, the system fails to reach its vision, “all citizens have the opportunity to achieve and maintain the highest level of health and wellbeing”, and the international objectives of the SDGs [16]. Iraq is the fifth largest oil producing country, yet, it has achieved little progress in its SDGs index score, from 60.05 in 2015 to 62.03 in 2022; these numbers show the significant impact of political, economic, and security instability on the country’s overall development [99,100]. An evaluation report of Iraq’s health system’s capacity to comply with the International Health Regulations (IHR) in 2019 showed that the system’s score was 47%, which was below the regional average of 60%; it also showed that the country’s abilities to prevent, detect, and respond to health emergencies were at 54%, 45%, and 47%, respectively [15].

Iraq achieved successes in different areas during the beginning of the pandemic, but the response did not reach an optimal level because of the political nature of the response, a weak decision-making process, a lack of authority to enforce public health measures, improper implementation of preventive measures, widespread conspiracy theories, and a lack of adequate infection prevention and control (IPC); the inadequacy of IPC measures led to high morbidity and mortality among HCWs [67], and the WHO reported 47,105 confirmed COVID-19 cases and 345 deaths among HCWs [44] in Iraq. The current pandemic has also shown that surveillance capabilities are not adequate as Iraq has been reported to be among the countries with significant data irregularities related to the current pandemic [101]. Additionally, the country heavily relies on the WHO surveillance system, the Early Warning Alert and Response Network (EWARN), which exists in only 7–8 governorates [23]. To build a comprehensive health information system, Iraq should significantly improve health policy and governance, data sources, institutional capacities, and mechanisms for data management [29]. For the provincial health facilities to become more proactive and responsive to their community’s health needs, district and governorate health facilities must have the capacity and authority to overcome weaknesses in the centralized health information system [6,38]. To further improve the health system’s responsiveness, capacity building in scientific research and pharmaceutical manufacturing, enhancing the administrative and operational components of Kimadia, and strengthening the Ministry of Health’s authority should be considered [38]. A national accreditation mechanism should also be established to set the minimum standard of care at PHCs. Accreditation has been shown to improve access to care, quality of care, and patient safety [102]. Additionally, if the PHCs are well-equipped and maintained, they could also serve as an additional resource for future health emergencies when additional space is needed.

Iran and Jordan, two upper-middle-income countries, provided more comprehensive social protection programs. In addition to the utility waivers, Iran provided financial support for its citizens in three rounds. The first round launched in March 2020 included four payments totaling USD 400 (given in four stages) and reached three million individuals with no fixed income; the second round included those without fixed income who were already registered in special social assistance programs. The second round reached 40 million people, and payments were distributed from December 2020 to March 2021. More targeted cash assistance was directed toward families with children who lived in four provinces that were hard hit by the pandemic. Additionally, Iran provided unemployment benefits to those who lost their jobs because of the pandemic [70]. Jordan expanded their cash transfer program to the most vulnerable households by providing monetary assistance for six months and increasing the number of households enrolled in the program. Additionally, the Government of Jordan provided temporary financial support for three months to daily wage workers; two cash assistance programs tailored to Syrian and Palestinian refugees were expanded in 2020. Moreover, a new COVID-19 stimulus package was announced in March 2021 to support underprivileged families with food vouchers during Ramadan. Jordan also provided unemployment benefits to those who lost their jobs because of the pandemic. Jordan differentiated its social protection programs from Iraq and Iran by recruiting youth to support their vaccination campaigns and providing childcare subsidies to working mothers [70]. Iraq should implement strategies that provide sustainable social protection programs and adopt policies for productive employment [103]. A registry system for daily wage workers should be implemented to identify those at risk of losing their income during crises.

Essential health services and routine vaccination programs are only boosted by supplementary vaccination activities or national vaccination campaigns, both of which were severely disrupted by the COVID-19 pandemic. Globally, the lack of PPE, limited healthcare workers, and travel restrictions were the main reasons for this disruption [104]. By December 2021, Iraq missed at least one VPDs campaign, leaving millions of children at risk of devastating diseases because of the limited import of vaccines, extended curfew, and the ban on international flights coming to the country [79]. Additionally, the fear of parents and children contracting an infection at a health facility might explain the decline in vaccination during the pandemic; however, research has shown that the risk of COVID-19 transmission is minimal if safety precautions are implemented properly [105]. For example, the Ethiopian Ministry of Health was able to vaccinate 1.5 million children against measles by establishing vaccination sites outdoor and in well-ventilated areas [105].

A successful GBV response requires a multi-sectoral and multi-level response, including Health, Mental Health and Psychosocial Support (MHPSS); legal aid; stable incomes for survivors; and an information management system to document, analyze, and share reports to inform policies and interventions [106]. Documented challenges to addressing GBV include inadequate and inconsistent funding; limited engagement of affected women; a lack of expertise in delivering services; and weak commitment from governments [106,107]. All these challenges have led to international organizations funding and taking leadership in addressing this issue [106]. The United Nations Population Fund (UNFPA) conducted some activities to mitigate the impact of COVID-19 on GBV. It developed a mobile application called SafeYou in Kurdistan to help women and girls at risk of GBV send their location to seven people in their social circle, including the police, if they feel unsafe. Additionally, the UNFPA also supported nine shelters that protect survivors of GBV [82]. Moreover, more collaboration has been established between the UNFPA and the government to enhance coordination at the governorate level. In the context of Iraq, only 5.5% of the GBV-requested funding was allocated to the country in 2014. Gender equality, which is the root cause of GBV, remains a major challenge in Iraq [100].

Violence toward HCWs is a public health issue associated with significant financial and human costs, which can also lead to legal consequences [108,109], and the current pandemic has exacerbated this threatening environment. Violence toward HCWs can be physical or psychological, and 25% percent of workplace violence occurs in the health sector [108]. A study observed that healthcare workers around the globe were more likely to experience harassment, bullying, and hurt than others during the pandemic [110]. The International Committee of the Red Cross (ICRC) reported on August 2020 that 67% of 611 violent incidents in medical facilities worldwide were directed against HCWs [111]. In Pakistan, 29 incidents against HCWs were reported in April–August 2020 [112]. The most cited reasons included mistrust in HCWs, belief in conspiracy theories, hospitals’ refusal to admit COVID-19 patients due to limited space, COVID-19 hospital policies, and the death of COVID-19 patients [110,112]. Many of these reasons are common in the context of Iraq [67,88,113].

The current pandemic has added more pressure on the health system and challenged the limited number of mental health professionals in Iraq [114]. The mental health system is not well-developed. A limited number of mental health facilities, a shortage of psychiatrists, stigma, and widespread misconceptions about mental health all contributed to the mental health impacts of the pandemic [115]. There are two mental hospitals and four mental health units in general hospitals in Baghdad, and another eight units in other governorates. There was one mental health professional for each 100,000 people in 2005 [116]. The National Mental Health Council (the first in the history of Iraq was established in 2003) expanded its activities and scope to become the National Council for Mental Health and Substance Misuse. It also contributed to the academic literature, increased its collaboration with the WHO, and established new partnerships with other non-governmental organizations to strengthen mental health services in Iraq [116]; however, this progress has been hindered by the aforementioned challenges Iraq has experienced. The COVID-19 pandemic has caused an increase in mental health disorders that will continue even after it ends.

To mitigate the impact of the pandemic on the health system in Iraq, some of evidence-based approaches are recommended:

- Immunization campaigns should be data-driven, people-centered, and flexible according to the local context. Additionally, enhancing VPDs surveillance and providing an adequate supply of PPE and vaccines are also important [117]. Moreover, planning an integrated vaccination campaign during COVID-19 vaccination is essential to avoid the accumulation of individuals with immunity gaps, as children might grow out of the target age for specific vaccines [105].
- Empowering girls and women by providing equal opportunities in the educational, economic, and political sectors; inclusion of GBV risk mitigation strategies as part of the COVID-19 response plan [118,119]; capacity building; cross-sector collaboration at all levels; scaling up of some of the local initiatives to other regions in Iraq; and increasing funding to GBV programs.
- Addressing violence against HCWs through different approaches, such as teaching de-escalation techniques; improving communication skills; limiting the number of patients’ visitors or restricting their movement within a health facility; implementing structural changes to the medical units; reducing waiting times; improving the quality of care; increasing the availability of medicines; and enhancing security and preventing the carrying of weapons inside health facilities [88,120,121].
- The implementation and expansion of telemedicine to offer mental health services, as evidence shows that telemedicine increases access to care; reduces travel and waiting times; and offers additional security measures to mental health professionals [122]. Additionally, the availability of digital applications can be used to screen for mental health disorders. The expansion and inclusion of mental health services at PHCs is another option. Mental health first aid training to HCWs, community engagement to raise awareness about mental health, and educational campaigns to normalize mental health should be considered. HCWs’ mental wellbeing should be a priority; some of the recommended strategies include setting health policies that support them financially and psychologically, such as decreasing shift hours to reduce exposure to COVID-19 patients, providing adequate PPE, ongoing education and workshops, and providing mental health resources [91,122].

### Limitations

The study is a qualitative paper based on a search of the published literature. This paper is limited by the number of databases used as the authors might have missed some of the literature that was not part of these three databases. Additionally, the search was based on the literature and reports written in English, so the exclusion of Arabic-written articles and reports might have occurred, resulting in bias and limited generalizability. Moreover, the term global public health used in the search was broad. While authors used different text words to mitigate the impact of this concept, the impact of using this term might not be known without conducting a new search with a more precise concept. The authors also did not search through the reference list of the included articles. Future studies should overcome some of the limitations reported in this study.

## 5. Conclusions

Iraq’s health system—influenced by decades of war, sanctions, and conflicts—has adapted an emergency rather than a proactive approach. With the current structure and resources, the system is unable to face the current pandemic or future health emergencies of similar magnitude without compromising the quality and quantity of health services. While the focus is on mitigating the impact of the pandemic, which may persist beyond the immediate pandemic, strengthening the health system should be the long-term goal. Coordinated efforts should be directed toward improving the six building blocks of a health system through strategic planning and capacity building, with the priority of optimizing the health information system and leadership/governance, as these provide the basis for health policies and regulations for all the other blocks. Iraq has made some progress in improving the health outcomes of its population, and this progress can be expedited and scaled up with proper leadership.

## Data Availability

Not applicable.

## Appendix A. Details about the Search Methods Used in This Article

**Table A1:** The research question and databases used in this paper.

Research Question	Databases	Limits
Current Impact and Long-Term Influence of the COVID-19 Pandemic on Global and Public Health, Public Policy, and Healthcare Systems: Socio-Economic Aspects—Iraq	PubMed Cochrane World Health Organization	

**Table A2:** Database: PubMed.

	Concept: COVID-19	Concept: Iraq	Concept: Global Public Health
**Thesaurus Terms/Subheadings**	“SARS-CoV-2” [MeSH] OR “COVID-19 Vaccines” [Mesh] OR “COVID-19”[Mesh] OR	“Iraq”[MeSH] OR	“Global Health” [MeSH] OR “Public Health”[MeSH] OR “Public Policy” [MeSH] OR “Delivery of Health Care”[Mesh] OR
**Textwords**	“SARS Coronavirus 2” OR “Coronavirus 2, SARS” OR “Coronavirus disease 2019 virus” OR “2019 Novel Coronavirus” OR “2019 Novel coronaviruses” OR “Coronavirus, 2019 Novel” OR “SARs-CoV-2 Virus” OR “SARS CoV 2 Virus” OR “SARS-CoV-2 Viruses” OR “Virus, SARS-CoV-2” OR “2019-nCoV” OR “COVID-19 virus” OR “COVID-19 Virus” OR “COVID 19 Virus” OR “COVID-19 Viruses” OR “Virus, COVID-19” OR “COVID-19” OR “COVID-19 vaccine” OR “COVID-19 cases” OR “COVID-19 deaths” OR “COVID-19 death” OR “COVID-19 timeline”	Iraq	“Global Health” OR “Public Health” OR “Public Policy” OR “Healthcare systems” OR “System, health care” OR “Health care system” OR “Health care systems” OR “Systems, Health Care” OR “Systems healthcare” OR “Census” OR “Population” OR “Geography” OR Demography OR Economy OR “Socioeconomic distribution” OR “Vector Ecology OR “Pandemic Response” OR “Pandemic Preparedness” OR “Pandemic Crises” OR “Anti-Science” OR “Anti-Vaccination” OR “Epidemiological curve” OR “Community strategies” OR “Social distancing” OR “Government Intervention” OR “Nonphamaceutical interventions” OR Vaccines OR “Testing capacity” OR Underreporting

Line 1: “SARS-CoV-2”[MeSH] OR “COVID-19 Vaccines”[Mesh] OR “COVID-19”[Mesh] OR “SARS Coronavirus 2” OR “Coronavirus 2, SARS” OR “Coronavirus disease 2019 virus” OR “2019 Novel Coronavirus” OR “2019 Novel coronaviruses” OR “Coronavirus, 2019 Novel” OR “SARS-CoV-2- Virus” OR “SARS CoV 2 Virus” OR “SARS-CoV-2 Viruses” OR “Virus, SARS-CoV-2” OR “2019-nCoV” OR “COVID-19 virus” OR “COVID-19 Virus” OR “COVID 19 Virus” OR “COVID-19 Viruses” OR “Virus, COVID-19” OR “COVID-19” OR “COVID-19 vaccine” OR “COVID-19 cases” OR “COVID-19 deaths” OR “COVID-19 death” OR “COVID-19 timeline”

Results: 253,404

Line 2: “Iraq”[MeSH] OR Iraq

Results: 19,096

Line 3: Line 1 + Line 2

Results: 591

Line 4: “Global Health”[MeSH] OR “Public Health”[MeSH] OR “Public Policy” [MeSH] OR “Delivery of Health Care”[MeSH] OR “Global Health” OR “Public Health” OR “Public Policy” OR “Healthcare systems” OR “System, health care” OR “Health care system” OR “Health care systems” OR “Systems, Health Care” OR “Systems healthcare” OR “Census” OR “Population” OR “Geography” OR Demography OR Economy OR “Socioeconomic distribution” OR “Vector Ecology OR “Pandemic Response” OR “Pandemic Preparedness” OR “Pandemic Crises” OR “Anti-Science” OR “Anti-Vaccination” OR “Epidemiological curve” OR “Community strategies” OR “Social distancing” OR “Government Intervention” OR “Nonphamaceutical interventions” OR Vaccines OR “Testing capacity” OR Underreporting

Results: 11,356,558

Line 5: Line 3 + Line 4

Results: 379

**Table A3:** Database: Cochrane Library.

	Concept: COVID-19	Concept: Iraq	Concept: Global Public Health
Thesaurus Terms/ Subheadings	“SARS-CoV-2”[MeSH] OR “COVID-19 Vaccines”[MeSH] OR “COVID-19”[MeSH] OR	“Iraq”[MeSH] OR	“Global Health”[MeSH] OR “Public Health”[MeSH] OR “Public Policy” [MeSH] OR “Delivery of Health Care”[Mesh]
Textwords	“SARS Coronavirus 2” OR “Coronavirus 2, SARS” OR “Coronavirus disease 2019 virus” OR “2019 Novel Coronavirus” OR “2019 Novel coronaviruses” OR “Coronavirus, 2019 Novel” OR “SARS-CoV-2- Virus” OR “SARS CoV 2 Virus” OR “SARS-CoV-2 Viruses” OR “Virus, SARS-CoV-2” OR “2019-nCoV” OR “COVID-19 virus” OR “COVID-19 Virus” OR “COVID 19 Virus” OR “COVID-19 Viruses” OR “Virus, COVID-19” OR “COVID-19” OR “COVID-19 vaccine” OR “COVID-19 cases” OR “COVID-19 deaths” OR “COVID-19 death” OR “COVID-19 timeline”	Iraq	Global Health OR Public Health OR Public Policy OR Healthcare systems “System, health care” “Health care system” “Health care systems” “Systems, Health Care” “Systems healthcare” “Census” OR “Population” OR “Geography” OR Demography OR Economy OR Socioeconomic distribution OR Vector Ecology OR Pandemic Response OR Pandemic Preparedness OR Pandemic Crises OR Anti-Science OR Anti-Vaccination OR Epidemiological curve OR Community strategies OR Social distancing OR Government Intervention OR Nonphamaceutical interventions OR Vaccines OR Testing capacity OR Underreporting

Line 1: “SARS-CoV-2”[MeSH] OR “COVID-19 Vaccines”[MeSH] OR “COVID-19”[MeSH] OR “SARS Coronavirus 2” OR “Coronavirus 2, SARS” OR “Coronavirus disease 2019 virus” OR “2019 Novel Coronavirus” OR “2019 Novel coronaviruses” OR “Coronavirus, 2019 Novel” OR “SARS-CoV-2- Virus” OR “SARS CoV 2 Virus” OR “SARS-CoV-2 Viruses” OR “Virus, SARS-CoV-2” OR “2019-nCoV” OR “COVID-19 virus” OR “COVID-19 Virus” OR “COVID 19 Virus” OR “COVID-19 Viruses” OR “Virus, COVID-19” OR “COVID-19” OR “COVID-19 vaccine” OR “COVID-19 cases” OR “COVID-19 deaths” OR “COVID-19 death” OR “COVID-19 timeline”

Results: 10,842

Line 2: “Iraq”[MeSH] OR Iraq

Results: 1146

Line 3: Line 1 + Line 2

Results: 21

Line 4: “Global Health”[MeSH] OR “Public Health”[MeSH] OR “Public Policy” [MeSH] OR “Delivery of Health Care”[Mesh] OR “Global Health” OR “Public Health” OR “Public Policy” OR “Healthcare systems” OR “System, health care” OR “Health care system” OR “Health care systems” OR “Systems, Health Care” OR “Systems healthcare” OR “Census” OR “Population” OR “Geography” OR Demography OR Economy OR “Socioeconomic distribution” OR “Vector Ecology OR “Pandemic Response” OR “Pandemic Preparedness” OR “Pandemic Crises” OR “Anti-Science” OR “Anti-Vaccination” OR “Epidemiological curve” OR “Community strategies” OR “Social distancing” OR “Government Intervention” OR “Nonphamaceutical interventions” OR Vaccines OR “Testing capacity” OR Underreporting

Results: 634,613

Line 5: Line 3 + Line 4

Results: 11

Date: 2 June 2022.

World Health Organization: COVID-19 AND iraq AND Anti-Vaccination OR Epidemiological curve OR Community strategies OR Social distancing OR Government Intervention

https://search.bvsalud.org/global-literature-on-novel-coronavirus-2019-ncov/?output=site&lang=en&from=0&sort=&format=summary&count=50&fb=&page=1&skfp=&index=&q=covid-19+AND+iraq+AND+Anti-Vaccination+OR+Epidemiological+curve+OR+Community+strategies+OR+Social+distancing+OR+Government+Intervention&search_form_submit= (accessed on 2 June 2020)

Results: 59

Total results from all databases: 390

Total results after deduplication + filter for “COVID-19 Global Health”: 154
